# Combination Therapy With Fingolimod and Neural Stem Cells Promotes Functional Myelination *in vivo* Through a Non-immunomodulatory Mechanism

**DOI:** 10.3389/fncel.2019.00014

**Published:** 2019-02-05

**Authors:** Yuan Zhang, Xin-Yu Lu, Ze-Qin Ye, Bogoljub Ciric, Cun-Gen Ma, Abdolmohamad Rostami, Xing Li, Guang-Xian Zhang

**Affiliations:** ^1^Department of Neurology, Thomas Jefferson University, Philadelphia, PA, United States; ^2^National Engineering Laboratory for Resource Development of Endangered Crude Drugs in Northwest China, The Key Laboratory of Medicinal Resources and Natural Pharmaceutical Chemistry, The Ministry of Education, Shaanxi Normal University, Xi'an, China; ^3^Department of Neurology, Institute of Brain Science, Shanxi Datong University Medical School, Datong, China

**Keywords:** fingolimod, neural stem cells, myelination, oligodendrocytes, combination therapy

## Abstract

Myelination, which occurs predominantly postnatally and continues throughout life, is important for proper neurologic function of the mammalian central nervous system (CNS). We have previously demonstrated that the combination therapy of fingolimod (FTY720) and transplanted neural stem cells (NSCs) had a significantly enhanced therapeutic effect on the chronic stage of experimental autoimmune encephalomyelitis, an animal model of CNS autoimmunity, compared to using either one of them alone. However, reduced disease severity may be secondary to the immunomodulatory effects of FTY720 and NSCs, while whether this therapy directly affects myelinogenesis remains unknown. To investigate this important question, we used three myelination models under minimal or non-inflammatory microenvironments. Our results showed that FTY720 drives NSCs to differentiate into oligodendrocytes and promotes myelination in an *ex vivo* brain slice culture model, and in the developing CNS of healthy postnatal mice *in vivo*. Elevated levels of neurotrophic factors, e.g., brain-derived neurotrophic factor and glial cell line-derived neurotrophic factor, were observed in the CNS of the treated infant mice. Further, FTY720 and NSCs efficiently prolonged the survival and improved sensorimotor function of *shiverer* mice. Together, these data demonstrate a direct effect of FTY720, beyond its known immunomodulatory capacity, in NSC differentiation and myelin development as a novel mechanism underlying its therapeutic effect in demyelinating diseases.

## Introduction

Inflammatory demyelination and axonal damage in the central nervous system (CNS) are pathological hallmarks of the chronic stage of multiple sclerosis (MS), an autoimmune demyelinating disorder that leads to severe neurological dysfunction (Plemel et al., 2017; Scolding et al., 2017). Although considerable progress has been made in MS therapies to reduce disease severity and progression, these therapies are only partially effective in the acute, but not the chronic state of disease (Bramow et al., 2010; Bible, 2013). This may be because they are mainly immunomodulatory but lack the neuroregenerative capacity to repair already damaged CNS tissue (Plemel et al., 2017). One major goal in therapeutic repair is the remyelination of demyelinated axons. Because remyelination is a natural consequence of oligodendrocyte differentiation, it involves promoting oligodendrocyte precursor cells (OPCs) to differentiate into mature oligodendrocytes in demyelinated lesions, thereby ensheathing axons with new myelin. Thus, a therapeutic strategy that has both immunomodulatory and (re)myelination effects is required to overcome the deficiency of current MS treatments (Grossman et al., 2017).

Fingolimod (FTY720, Gilenya), a lipophilic sphingosine 1-phosphate (S1P) analog, is the first oral therapy for MS (Brinkmann et al., 2010). The primary effect of FTY720 is blocking lymphocyte egress from lymphoid tissues, thereby reducing CNS infiltration (Aktas et al., 2010). Apart from its well-known immunomodulatory actions, there is evidence that FTY720 and related compounds could exert direct effects on the CNS, because of its lipophilic nature, as well as the expression of S1P receptors on neuroglial cells (Hunter et al., 2016). There is thus the possibility that FTY720 might exert additional beneficial effects on MS disease progression independent of its effect on lymphocyte sequestration in lymph nodes (Miron et al., 2008b). Our previous study (Zhang et al., 2017) demonstrated that FTY720, at nanomolar concentrations, effectively protected neural stem cell (NSC) survival and enhanced their development into mature oligodendrocytes *in vitro*. *In vivo* studies in an animal model of MS, experimental autoimmune encephalomyelitis (EAE), have shown significantly enhanced effects of combination therapy with FTY720 and NSCs, compared to either one alone, on clinical amelioration, axon preservation, neural cell survival, and transplanted NSC differentiation into mature oligodendrocytes, all of which are frequently linked to improved remyelination and CNS repair processes.

Despite extensive studies, however, some important issues remain to be clarified. On the one hand, the neurologic recovery of chronic EAE mice treated with FTY720 and NSCs might be secondary to their immunomodulatory effects. On the other hand, there are reports showing that FTY720 did not induce remyelination in cuprizone- and lysophosphatidylcholine-induced demyelination models. Therefore, whether the combination therapy with FTY720 and NSCs could induce myelination directly in a non-chemical, minimal or non-inflammatory CNS microenvironment has not yet been studied. The potential of FTY720 to enhance an NSC-based therapy for MS, as well as other demyelinating disorders, remains to be determined.

To address this issue, here we sought to investigate the role of the FTY720/NSC combination during the myelination process in three myelination models with minimal or non-immune cell involvement, including organotypic slice culture, postnatal oligodendrocyte maturation, as well as the *shiverer* mouse model. The combination therapy with FTY720 and NSCs was effective in promoting precocious myelination in organotypic slice cultures, and *in vivo* in early postnatal mouse pups. Treatment with both FTY720 and NSCs, but not with FTY720 or NSCs alone, significantly increased differentiation of transplanted NSCs into oligodendrocytes in hypomyelinated shiverer brain. The mechanism underlying the beneficial effect of FTY720 on NSC directional differentiation and myelination has also been addressed.

## Materials and Methods

### Animals

All experimental procedures and protocols were approved by the Thomas Jefferson University Institutional Animal Care and Committee and were performed in accordance with the approved institutional guidelines and regulations. C57BL/6, C3H, or *shiverer* mice (C3H background) were purchased from Jackson Laboratory (Bar Harbor, ME). Mice, 1–3 days after birth, were used in all *in vivo* experiments.

### NSC Generation

NSCs were generated from the SVZ area of C57BL/6 or C3H mice (Jackson Laboratory, Bar Harbor, ME), 6–8 weeks of age, and transduced with GFP as described previously (Li et al., 2016). GFP is used for tracing transplanted NSCs in brain slice cultures. NSCs without GFP expression at passages 5–15 were used in *in vivo* myelination experiments.

### Antibodies

Primary antibodies used for these studies were specific for: myelin basic protein (MBP, Abcam), neuron (NeuN, Abcam), neurofilament (NFH, Abcam), glial fibrillary acid protein (GFAP, Abcam), adenomatous polyposis coli/CC1 (Millipore). Appropriate fluorescent secondary antibodies were used (Alexa Fluor, Invitrogen).

### Organotypic Slice Culture Myelination Analysis

Organotypic slice cultures were prepared from the forebrain of C57/Bl6 mouse pups as previously described (Miron et al., 2010). Following 3-day *ex-vivo* culture to allow debris clearance and dissection recovery, dissociated single NSCs (2 μl, ~5 × 10^4^ cells/slice) were transduced with lentivirus expressing GFP and were pipetted directly onto brain slices, which had been pre-treated with cytosine arabinoside to prevent endogenous myelination (Nishimura et al., 1985). Meanwhile, FTY720 (1 nM) was diluted in culture media and replaced daily. For myelin maintenance studies, slices transduced with or without NSCs were treated for the subsequent 14 days with FTY720. Slices that were cultured with both NSCs and FTY720 served as a co-treatment group, those that received either NSCs or FTY720 alone served as single-treatment controls, and those that were incubated with the same volume of culture medium served as a sham-treated control. Slices were cultured for 2 weeks and fixed in 4% paraformaldehyde (PFA, wt/vol) for 1 h and blocked in 5% normal horse serum (GIBCO) and 0.3% Triton-X-100 (Fisher Scientific) for 3 h at room temperature. Primary antibodies diluted in block solution were applied and washed out with PBS 3 times after 48-h incubation at 4°C. Slices were then incubated with species-specific secondary antibodies for 1 h at room temperature, followed by washing with PBS 3 times. Immunofluorescence controls were routinely prepared by omitting primary antibodies. Nuclei were stained with DAPI. Slides were covered with mounting medium (Vector Laboratories, Burlingame, CA, USA) and kept at room temperature for further analysis.

### Myelination During Postnatal Development

C57Bl/6 mice, 3 days after birth, were used in myelin development experiments as previously described (From et al., 2014). NSCs were prepared for transplantation as described for *in vitro* culture. Male littermates of similar weights were randomly enrolled in the following treatment groups: (1) NSC single-treated group: dissociated single NSCs (2.0 × 10^5^ cells/mouse) were injected i.c.v. at day 3 postnatal under anesthesia on ice as described (Zhang et al., 2017), and PBS injected i.p. daily. (2) FTY720 single-treated group: FTY720 at a dose of 0.3 mg/kg daily was injected i.p. daily, and PBS injected i.c.v. at day 3 postnatal. (3) Combination of FTY720 and NSC treatment group: NSCs were injected i.c.v. at day 3 postnatal with FTY720 i.p. (0.3 mg/kg daily) from the day of cell injection. (4) PBS-injected i.c.v. and i.p. in the same way as described above served as control. Transverse sections of spinal cord (L4-L5 segments) were harvested at day 14 postnatal and processed for immunohistochemistry or RNA extraction. The myelinated axons (NFH^+^MBP^+^) were quantified within 5 random standard 500 μm^2^ fields in the white matter of the ventral lumbar spinal cord for each mouse. Immunohistochemistry was performed using different antibodies following procedures described previously (Zhang et al., 2017). At days 20–21 postnatal, behavioral assessments (open-field test and rotating rod test) of littermate mice from four different groups were performed based on published protocols (From et al., 2014). Data were collected from three separate mouse litters because variability between litters has been reported.

### Myelination of *Shiverer* Mice

Newborn double-homozygous *shiverer* (*shi/shi*) mice (The Jackson Laboratory, Bar Harbor, ME) were implanted with dissociated single NSCs, with or without FTY720 from the day of cell injection. NSCs were transplanted bilaterally in the corpus callosum of newborn *shiverer* mice (2.0 × 10^5^/mouse) starting from 0 to 2 days of age, following a protocol using hematopoietic stem cell transplantation (Windrem et al., 2004). *Shiverer* mice that received NSCs, or FTY720 (injected i.p. at 0.3 mg/kg daily) alone served as single-treatment controls, and mice that received the same volume of PBS served as a sham-treated control. The mice were then returned to their mothers, and allowed to develop normally, with weaning at 28 days and small group housing thereafter. At 3 months of age, mice were anesthetized, perfused, and brains were isolated for FluoroMyelin staining, an indicator of mature myelin formation. FluoroMyelin green fluorescent myelin stain was performed following the manufacturer's instructions (ThermoFisher Scientific). Kaplan-Meier analysis was used to assess the survival of *shiverer* mice in different groups, as described (Shinkai et al., 1992).

### Real-Time RT-PCR

Total RNA was extracted from spinal cords using RNeasy® Plus Mini Kit (QIAGEN, Valencia, CA) according to the manufacturer's instructions. Reverse transcription was conducted using QuantiTect® Reverse Transcription Kit (Qiagen, Valencia, CA). Real-time PCR was performed using the Custom RT2 Profiler PCR Array (Qiagen, Valencia, CA) for mouse growth factors (Cat. no. 330231 PAMM-041ZA), and detection was performed using the ABI Prism® 7500 Sequence Detection System (Applied Biosystems, Foster City, CA).

### Immunohistochemical Analysis

Results were visualized by fluorescent microscopy (Nikon Eclipse E600; Nikon, Melville, NY) or confocal microscopy (Zeiss LSM 510; Carl Zeiss, Thornwood, NY). Quantitative image analysis was performed using ImageJ (NIH) and ImagePro (Media Cybernetics). To quantify FluoroMyelin, MBP, NFH, or GFAP expression, pixel intensity immunofluorescence was measured. All slides were immunostained with MBP, NFH or GFAP antibodies, and all digital images were acquired using the same exposure parameters. Pixel intensity of different markers was measured in disease lesions in the same image size using ImagePro software (Media Cybernetics). The mean pixel intensity was calculated from 20 to 25 images per mouse, 5 mice per group were evaluated. To quantify CC1^+^, NeuN^+^, or transplanted GFP+ cells within the slices, 5–10 digital photographs were taken using the same exposure parameters from each section, and 5 mice per group were evaluated. Cell counter of ImagePro software (Media Cybernetics) was used to count cells, and mean numbers were used for analysis.

### Electron Microscopy

Mice were deeply anesthetized and perfused with 4% paraformaldehyde, 1.5% glutaraldehyde and 1 mM CaCl_2_ in 0.1 M cacodylate buffer. The spinal cords were exposed and fixed in the same solution at 4°C for 24 h. Samples were washed, post-fixed with 1% OsO_4_ in 0.1 M PBS (pH 7.4) for 2 h at room temperature, and subsequently dehydrated in graded ethanol series. Embedding was performed in TAAB resin. Sections, 1.0 μm thick, were cut, stained in toluidine blue (1%), and examined by light microscopy (E800, Nikon) for general histological assessment. Ultrathin sections (60–80 nm) were cut, viewed and photographed with a HT7700 (Hitachi) transmission electron microscope operated at 120 kV. Images were analyzed in Image-Pro for thickness of myelin sheath and g-ratio.

### Statistical Analysis

Statistical analyses were performed using GraphPad Prism 6 software (GraphPad, La Jolla, CA). Data are presented as mean ± SD. When comparing multiple groups, data were analyzed by analysis of variance (ANOVA) with Tukey's multiple comparisons test. A significance criterion of *p* < 0.05 was used for all statistical analysis.

## Results

### FTY720 Drives Transplanted NSCs to Differentiate Into Oligodendrocytes and Promotes Myelination in an *ex vivo* Organotypic Slice Culture Model

**Figure 1:**
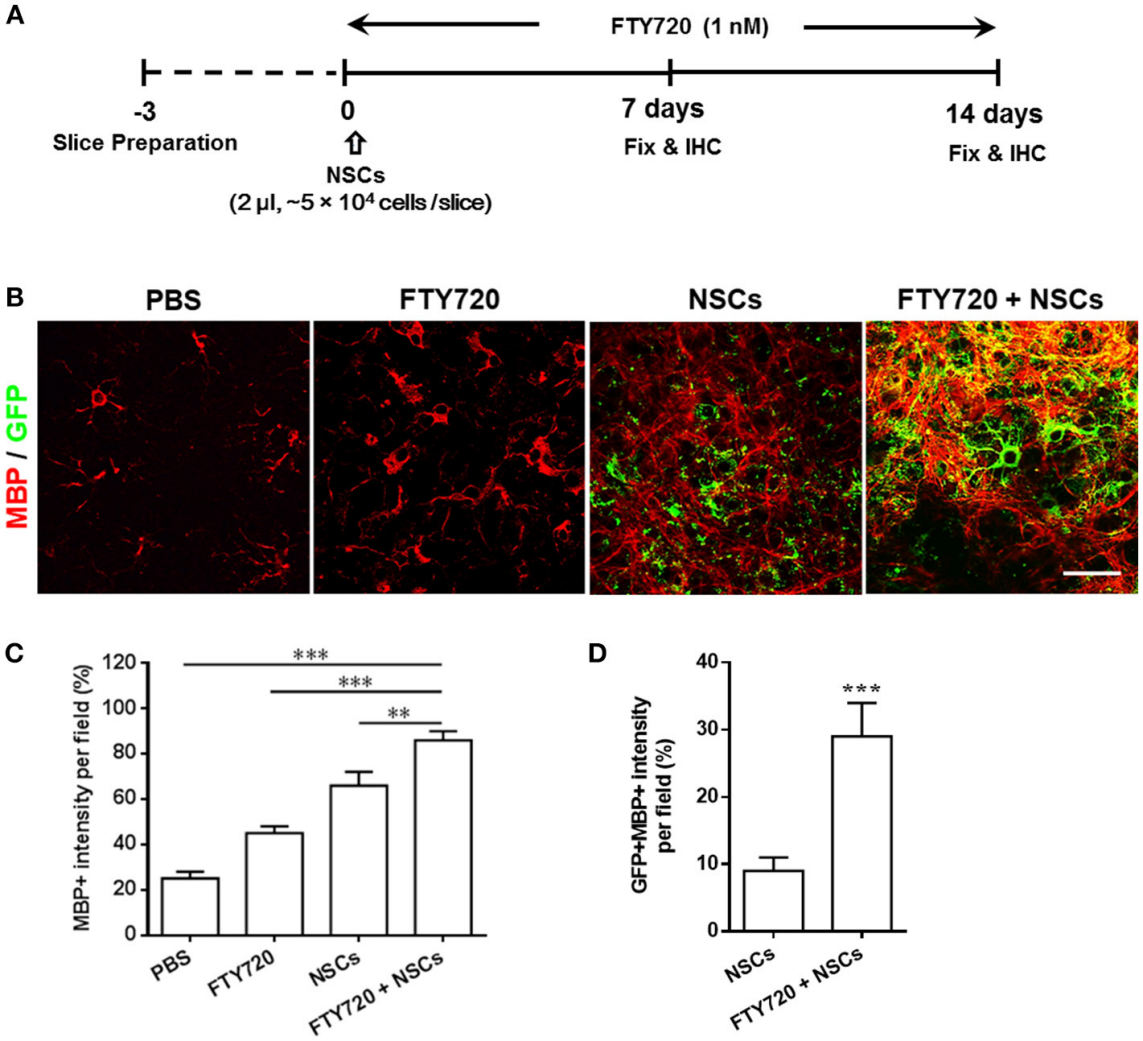
Co-treatment with FTY720 and NSCs promoted myelination in the brain slice culture model. **(A)** Schematic diagram illustrating experimental design followed throughout this study. Slices obtained from newborn (P0-3) C57/Bl6 mouse pups were grown for 3 days, and NSCs (2 μl, ~5 × 10^4^ cells /slice) and/or FTY720 (1 nM) were added for the subsequent 14 days, after which the slices were fixed and immunostained. **(B)** Representative confocal images displaying GFP + cells (green; NSCs) and MBP (red) immunoreactivity after 14 days of treatment. Scale bar = 20 μm. **(C)** MBP intensity was measured using Image-Pro. **(D)** GFP + MBP + intensity was measured using Image-Pro. Representative images are shown, and quantitative data refer to mean ± SD (*n* = 6 slices/group). ^**^p < 0.01, ^***^p < 0.001. One-way ANOVA with Tukey's multiple comparisons test and unpaired Student's *t*-test. One representative of 3 independent experiments is shown.

The beneficial effects of FTY720 on NSC differentiation into oligodendrocytes *in vitro* (Zhang et al., 2017) prompted us to investigate whether FTY720 could enhance myelination and myelin maintenance under nonpathological conditions in an *ex-vivo* culture of brain slices, which maintain the true three-dimensional structure of the tissue (Humpel, 2015). To this end, following 3 days of recovery from dissection in culture, dissociated single NSCs and/or FTY720 were added to mouse brain slices for the subsequent 2 weeks. Experimental design and treatment strategies are shown in Figure 1A. After 14 days, some exogenous GFP-positive NSCs were attached on the surface, or the edge of the slice, but others penetrated into the tissue slice structure, matured and myelinated (Figure 1B). Co-treatment with FTY720 and NSCs remarkably increased the area and intensity of MBP staining per field relative to sham-treated control (~2.86 fold over control, *p* = 0.0005), while a single treatment (either NSCs or FTY720) resulted in a slight, but significantly greater, improvement in myelin maintenance than in the sham-treated control group (Figure 1C). Interestingly, while NSCs (GFP^+^) without FTY720 injection did not express MBP, a large proportion (~29%) of those with FTY720 did so, indicating that FTY720 not only enhanced brain tissue myelination, but also promoted NSC differentiation into mature oligodendrocytes (Figure 1D).

**Figure 2:**
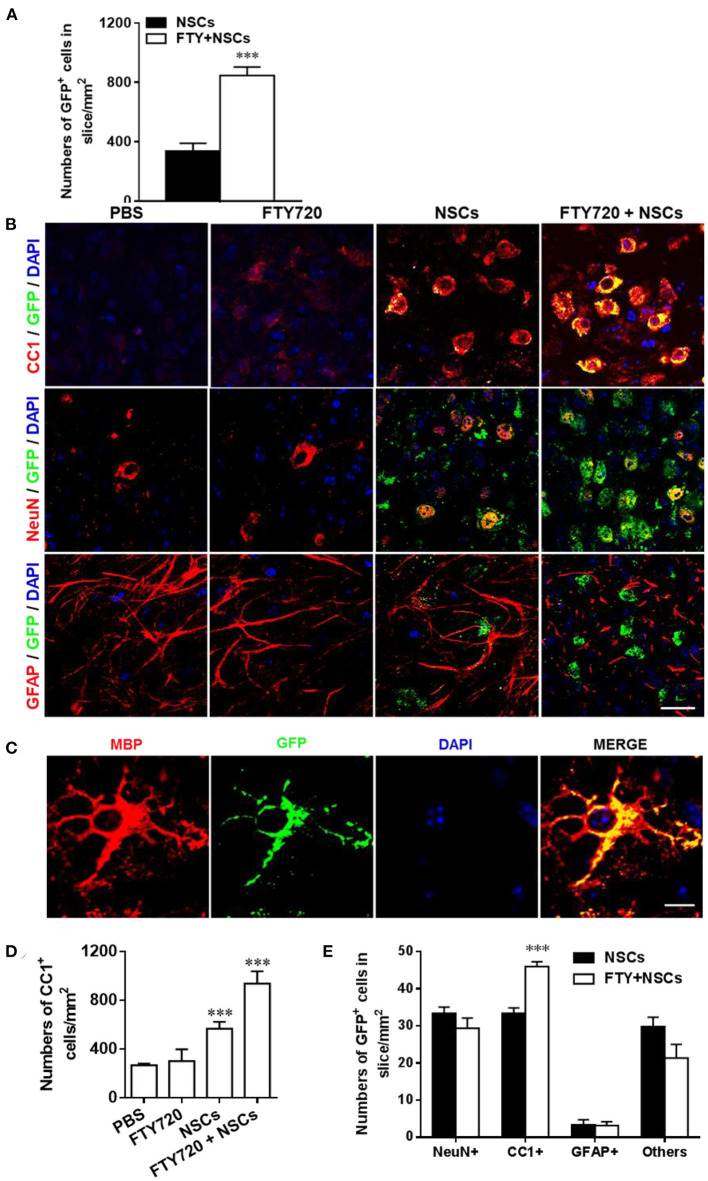
Impact of FTY720 plus NSC treatment on neural cell lineages in brain slice culture model. Brain slices obtained from newborn (P0-3) C57/Bl6 mouse pups were grown for 3 days in culture, and NSCs (2 μl, ~ 5 × 10^4^ cells /slice) and/or FTY720 (1 nM) were added for the subsequent 7 days; the slices were then fixed and immunostained. **(A)** Quantitative analysis of the number of transplanted NSCs (GFP+) in brain slices. **(B)** Immunofluorescence images of brain slices. Cells co-labeled with GFP and neural specific markers (red) were identified as differentiated cells derived from NSCs (yellow); cells positive only for neural-specific markers (red) were endogenous cells. CC1^+^: oligodendrocytes, NeuN+: neurons, GFAP+, astrocytes. Scale bar = 20 μm. **(C)** High-magnification confocal images show that GFP (green) was highly colocalized with the oligodendrocyte marker MBP (red). Scale bar = 10 μm. **(D)** Quantification of total CC1^+^ cell numbers. **(E)** Quantitative analysis of differentiation of transplanted NSCs in the CNS as shown in **(B)**. Symbols represent mean ± SD; *n* = 10 random areas per group. ^***^p < 0.001. One-way ANOVA with Tukey's multiple comparisons test and unpaired Student's *t*-test. One representative of 3 independent experiments is shown.

We next investigated the effects of FTY720 on the fate of NSCs in the cultures by labeling them with neural cell-specific antibodies after 7 days of FTY720 treatment. A significantly increased number of NSCs (GFP^+^) was observed in FTY720 and NSC co-treated wells compared to the single NSC treatment group. Quantitative analysis showed that approximately 847 ± 59 GFP^+^ cells/mm^2^ were found in the brain slice of the co-treatment group, while approximately 337 ± 56 GFP^+^ cells/mm^2^ were found in slices treated with NSCs alone (Figure 2A). Co-localization of GFP^+^ and neural-specific markers revealed the differentiation of transplanted NSCs into CC1^+^ mature OLGs and NeuN^+^ neurons (Figure 2B). Rare GFP^+^ NSC colocalizing with GFAP was observed in mice treated with NSCs alone or in combination with FTY720 (Figures 2B,E), while the co-treatment globally reduced the number and branches of astrocytes compared to other groups (Figure 2B). High-magnification confocal images further confirmed that the grafted NSCs differentiated into MBP^+^ mature oligodendrocytes with membrane elaboration (Figure 2C). Further, the numbers of CC1^+^ OLGs as well as transplanted CC1^+^ GFP^+^ OLGs in the NSC plus FTY720 co-treated group were significantly higher than in those treated with NSCs alone (Figures 2D,E). The numbers of NeuN^+^ GFP^+^ neurons were similar (Figure 2E). Together, these results indicate that FTY720 not only enhanced formation of myelin sheaths in brain slice cultures, but also promoted survival of NSCs, adding and driving their differentiation into mature oligodendrocytes, thus further promoting myelination.

### The Effects of FTY720 And/Or NSC Treatment on CNS Myelinogenesis

**Figure 3:**
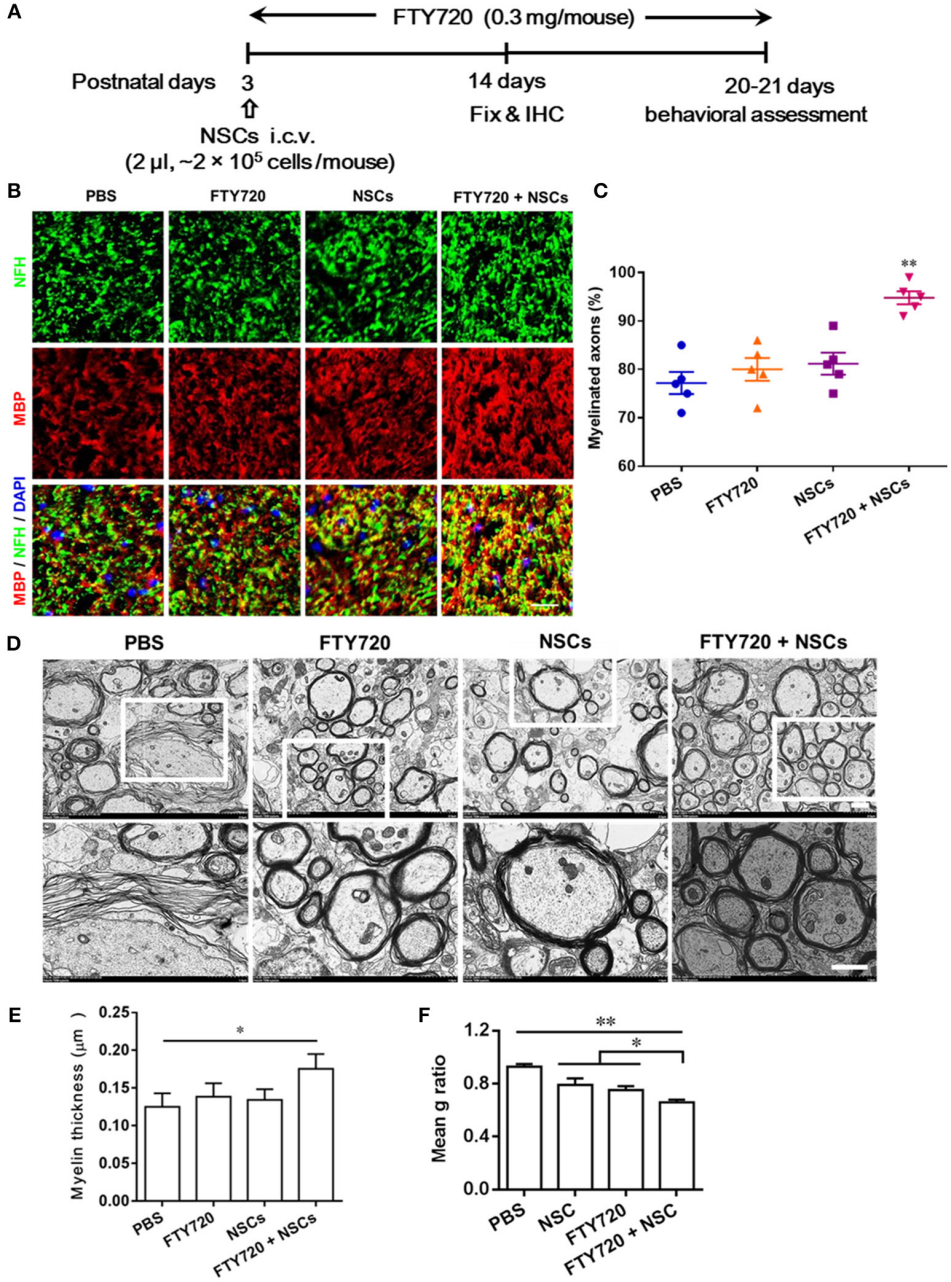
Co-treatment with FTY720 and NSCs promoted myelination in the CNS of newborn mice *in vivo*. **(A)** Schematic diagram illustrating experimental design followed throughout this study. Mice were i.c.v. injected with NSCs (2 μl, ~2 × 10^5^ cells/mouse) at day 3 postnatal, then injected daily with FTY720 i.p. (0.3 mg/mouse) or with PBS from day 3 postnatal. Spinal cords were harvested at day 14 postnatal, and stained with antibodies to MBP for myelin and to NFH for axonal fibers. **(B)** Representative confocal images displaying NFH (green) and MBP (red) immunoreactivity. Scale bar = 10 μm. **(C)** Quantification of myelinated axons from the total number of axons in 5 mouse litters each group, performed within 5 random standard 500 μm^2^ fields in the white matter of the ventral lumbar. **(D)** Representative electron micrographs of transverse lumbar sections of the ventral region. Scale bar = 1.0 μm. **(E)** Quantification of the mean thickness of myelin sheaths. **(F)** Mean g ratio (axon diameter divided by the entire myelinated fiber diameter) was determined using Image-Pro Plus software. Symbols represent mean ± SD. ^*^p < 0.05, ^**^p < 0.01. One-way ANOVA with Tukey's multiple comparisons test and unpaired Student's *t*-test.

Myelinogenesis, the establishment of the myelin sheath in the mammalian nervous system, occurs predominantly postnatally within the first 3 weeks in rodents (Hughes and Appel, 2016). To investigate whether FTY720 or/and NSCs can affect myelinogenesis and oligodendrogenesis in the developing nervous system under nonpathological conditions, myelination extent was quantified in the spinal cord of newborn mice at day 14 postnatal, when myelination was not complete (Figure 3A). Up to 77% of the myelinated axons (co-localization of NFH and MBP) were observed in PBS-injected mice, while ~81% and 80% myelinated axons were detected in mice treated with FTY720 or NSCs alone, respectively, while there was no obvious increase in myelination compared with the PBS-injected littermates. Notably, in the group with FTY720 and NSC co-treatment, the extent of myelinated axons was remarkably increased compared with the PBS-injected littermates and those treated with either FTY720 or NSCs alone (*p* < 0.05; Figures 3B,C).

To further evaluate the effects of FTY720 or/and NSCs on CNS myelination, ultrastructural EM was applied to determine myelin layer thickness, as well as axonal size (Figures 3D–F). Consistent with the findings obtained by immunohistochemistry, thicker myelin sheath was found in the FTY720 and NSC co-treatment group compared with the PBS-injected littermates (Figures 3D,E). This was also manifested in the averaged g-ratio (the ratio between diameter of axon and total fiber) obtained at postnatal day 14 (Figure 3F). These results clearly indicate an accelerated myelin development induced by FTY720 and NSCs in the CNS.

**Figure 4:**
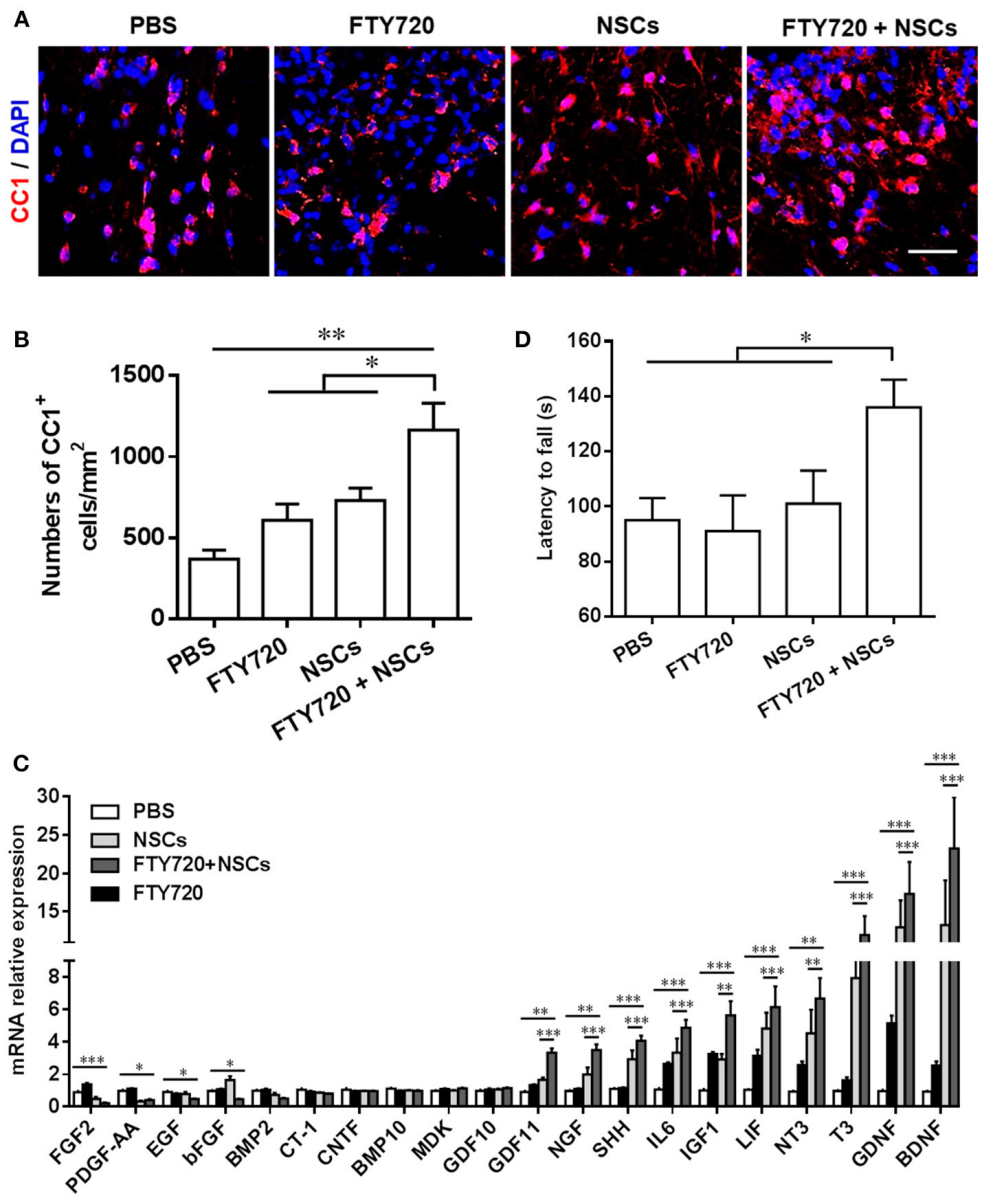
The effect of co-treatment with FTY720 and NSCs on the number of mature oligodendrocytes in the spinal cord of newborn mice. Spinal cords of mice described in Figure 3 were harvested at day 14 postnatal, and lumbar sections were stained for newly matured oligodendrocyte marker CC1. **(A)** Representative confocal images displaying CC1^+^ (red) immunoreactivity at day 14 postnatal. Scale bar = 10 μm. **(B)** Quantification of total CC1^+^ cell numbers. **(C)** Expression of growth factor genes in brain tissues was determined using Custom RT2 Profiler™ PCR Array Mouse Growth Factors (Qiagen, Valencia, CA). Relative expression was calculated by –ΔΔCt values from triplicate of PCR (*n* = 3; mean + SEM, Student's *t*-test; normalized to PBS group). **(D)** The effect of postnatal FTY720 or/and NSC administration on the performance of newborn mice in accelerating rotating rod test was examined at day 21 postnatal. Symbols represent mean ± SD. ^*^p < 0.05, ^**^p < 0.01, ^***^p < 0.01. One-way ANOVA with Tukey's multiple comparisons test and unpaired Student's *t* test.

The numbers of CC1^+^ cells, the newly matured oligodendrocytes, were then quantified in the spinal cord at day 14 postnatal. Consistent with the myelination results shown in Figure 3, the numbers of CC1^+^ oligodendrocytes were significantly increased in both the FTY720 and NSCs-single treated groups (~1.25 and 1.33-fold higher), vs. PBS-treated control littermates (Figures 4A,B). In the NSC and FTY720 co-treated group, a ~2.75-fold increase in the number of CC1-expressing cells was detected at day 14 postnatal, in comparison to the PBS-injected littermates, which was significantly higher than in the other three groups. These results indicate the combined effects of promoting myelinogenesis and oligodendrogenesis in the developing nervous system under nonpathological conditions.

We further studied the effects of postnatal administration of FTY720 and/or NSCs on the expression of several growth factors, given the essential role of these growth factors in CNS development (Acosta et al., 2013). As shown in Figure 4C, single- and co-treatment regulated the expression of several growth factors and neurotrophins, including BDNF, GDNF, T3, NT3, LIF, IGF1, IL6, SHH, NGF, GDF11, in varying degrees; while the fold change in the FTY720 and NSC co-treated group was most remarkable. The results indicate that single-NSC treatment may also induce a trophic effect *per se*, and that the co-treatment with FTY720, even though beneficial in myelination improvement, does not have a direct effect on this specific mechanism. Among the differentially expressed genes, the most robustly induced (~30-fold higher over PBS control) were brain-derived neurotrophic factor (BDNF) and glial cell line-derived neurotrophic factor (GDNF), important growth factors produced by CNS cells, promoting NSC survival and improving neurogenesis. Collectively, FTY720 and NSC treatment significantly promoted myelination in the CNS, most likely through creating a beneficial environment, e.g., induction of neurotrophic factors.

Although immunohistochemical studies showed significant differences, mice in different groups demonstrated no gross morphological abnormalities, bred efficiently, and demonstrated comparable body weights to controls throughout development (data not shown). To determine if the accelerated myelin development induced by FTY720 and/or NSC resulted in any advantage in sensor/motor function, we performed a behavioral assessment of littermate mice from four different groups at days 20–21 postnatal. An open-field test was performed to assess anxiety and voluntary locomotor activity at day 20 postnatal, and the balance and motor coordination of the mice were evaluated using a rotating rod test at day 21 postnatal. In the open-field test, mice in neither the single- nor the co-treatment group made significantly different stops compared with their PBS-injected littermates, indicating no difference in the anxiety indices among the four groups in this test (data not shown). In the rotating rod test at accelerating speed (Figure 4D), FTY720 and NSC co-treatment mice exhibited a significantly longer latency in falling off the rod than the other three groups of mice (an increase of 1.43-fold in the time they stayed on the rotating rod). These findings suggest the improvement brought about by FTY720 and NSC co-treatment in balance and motor coordination of the newborn mice.

### Transplanted NSCs Prolonged the Survival of *Shiverer* Mice, and FTY720 Significantly Enhanced This Effect

To further confirm the direct effect of FTY720 on the *in vivo* development of NSCs into myelinating cells, the *shiverer* mouse model was included. *Shiverer* mice have a spontaneous deletion of multiple exons in the gene encoding myelin basic protein (MBP), resulting in pronounced ataxia by 2–3 weeks of age, as well as the onset of fatal seizures by ~8–14 weeks (Windrem et al., 2008). Congenitally hypomyelinated *shiverer* mice fail to generate compact myelin and die by 18~21 weeks of age. Thus, any myelin produced by NSCs transplanted into a *shiverer* CNS can be unambiguously identified by positive immunohistochemical staining for MBP (Wang et al., 2013).

**Figure 5:**
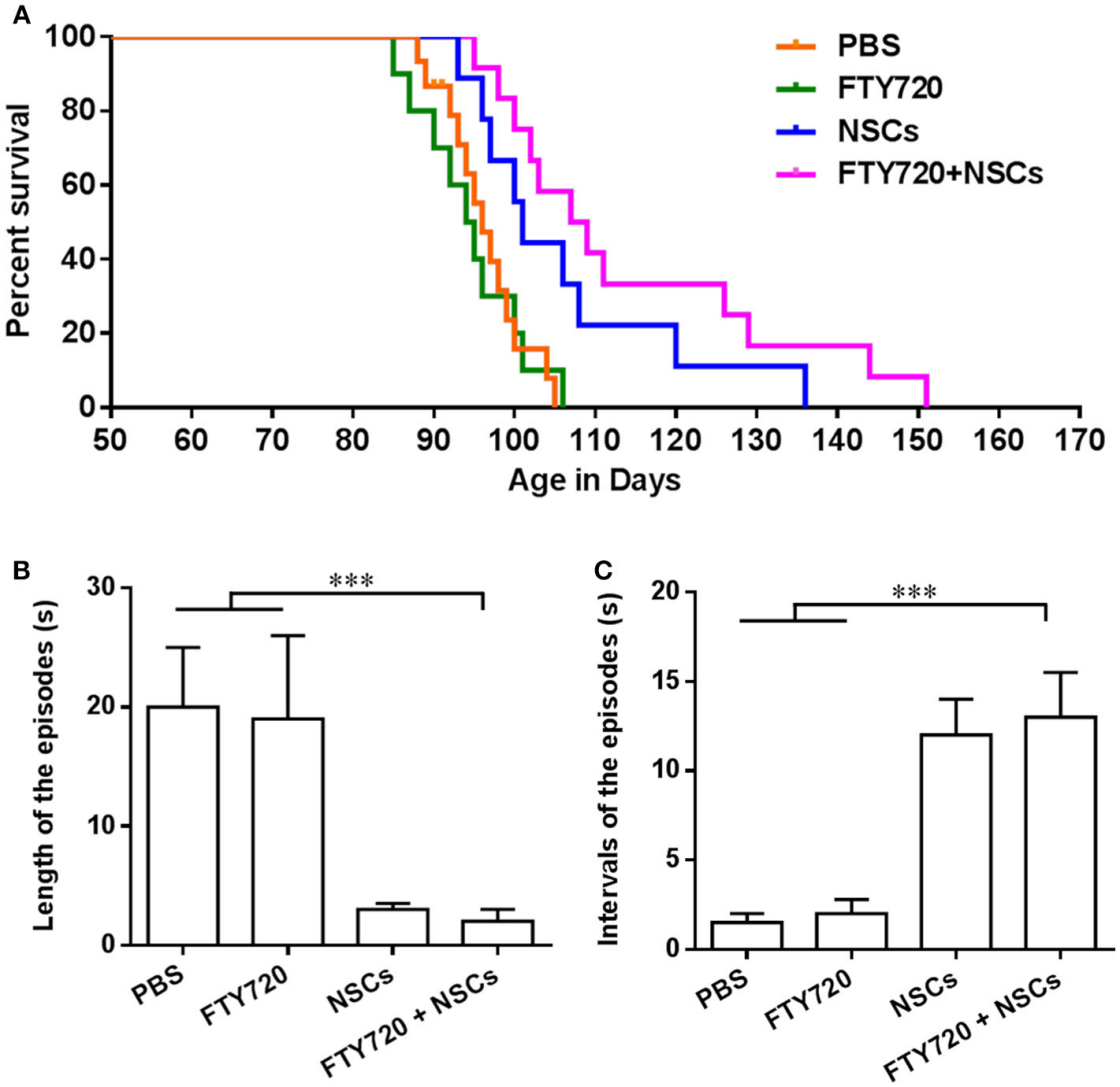
FTY720 in combination with transplanted NSCs improved neurological function and diminished seizures of *shiverer* mice. **(A)** Transplanted NSCs and FTY720-NSC co-treatment prolong the survival of shiverer mice. Newborn double-homozygous shiverer (*shi/shi*) mice were implanted i.c.v. with dissociated single NSCs (2.0 × 10^5^ cells/mouse), and injected i.p. with fingolimod (at 0.3 mg/kg daily) (*n* = 12) from the day of cell injection. Shiverer mice that received NSCs (*n* = 14), or fingolimod (*n* = 12) alone served as single-treatment controls, and mice that received the same volume of phosphate-buffered saline (PBS) served as a sham-treated control (*n* = 12). All these shiverer mice were maintained in small group housing and monitored daily until death. The Kaplan-Meier survival graph, plotting the percentage of each group alive in days, is shown. **(B)** Quantification of length of episodes. **(C)** Quantification of intervals of episodes. Symbols represent mean ± SD. ^***^p < 0.01. One-way ANOVA with Tukey's multiple comparisons test and unpaired Student's *t* test.

Predictably, untreated *shiverer* mice exhibited markedly impaired forward ambulation and frequent episodes of sustained seizures. Over a range of 85–106 days postnatally, all the PBS-treated and FTY720-treated *shiverer* mice died, with a median survival of 95.4 and 95.0 days, respectively. No obvious differences were observed between these two groups (Figure 5A). Animals transplanted with NSCs exhibited a significantly prolonged survival, with an average survival of approximately 105.8 days. Remarkably, the proportion of surviving *shiverer* mice was greater in the FTY720+NSC co-treated group than in the group engrafted with NSC only, with an average survival of 114.6 days.

### FTY720 in Combination With Transplanted NSCs Improved Neurological Function and Diminished Seizures of *Shiverer* Mice

*Shiverer* mice typically exhibit truncal instability and marked intention tremor, evident within a few weeks of birth, which becomes complicated by a progressive hindlimb weakness, such that by 95–105 days of age, they are severely impaired and manifest status epilepticus (Video 1) until they died. Given the substantially longer survivals noted in the FTY720-NSC co-treated *shiverer*, we then tested whether neurological function was improved after treatment. We noted that all groups of the *shiverer*, treated or untreated, deteriorated identically over the first few weeks after birth. However, up to 100 days, the point at which mice typically began to die, little difference was observed in the behavior of co-treated relative to NSC single-treated *shiverer* mice. All those mice survived the period spanning 120–150 days postnatally, and exhibited noticeable improvement in their neurological function manifested by diminished frequency of seizures and improved ambulation (Video 2). We also quantified the neurological improvements ~100 days of age (Figures 5B,C). *Shiverer* mice treated with NSCs alone as well as FTY720/NSC co-treatment exhibited a significantly shorter duration but longer intervals of episodes than the PBS or FTY720 single-treated groups of mice (Figures 5B,C). However, at this time point, no remarkable difference was observed in the neurological improvement of the co-treatment group relative to NSC single-treated shiverer mice (Figures 5B,C). Accordingly, NSCs or FTY720+NSC co-treated groups exhibited markedly improved neurological function, with decreased seizure incidence and improved mobility and self-care compared to mice treated with PBS or FTY720 alone.

### FTY720 in Combination With Transplanted NSCs Efficiently Myelinated the *Shiverer* Brain

**Figure 6:**
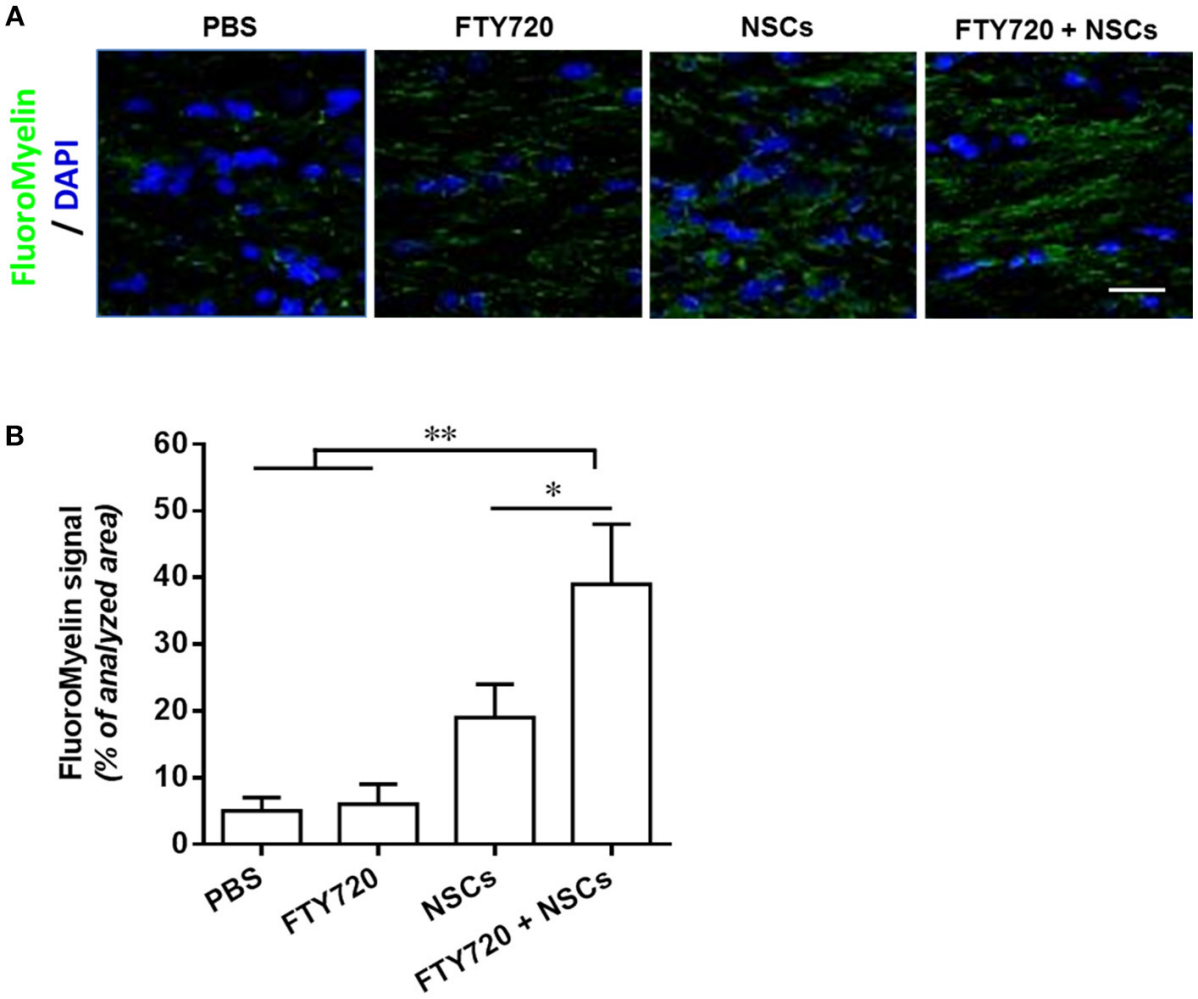
FTY720 in combination with transplanted NSCs efficiently myelinated the shiverer brain. In separate experiments, mice were treated as described in Figure 5, and brains were harvested at day 90 postnatal for immunostaining. **(A)** Confocal micrographs showing FluoroMyelin staining in the midline of the corpus callosum of shiverer mice treated with NSCs and/or FTY720. **(B)** Averaged values (mean ± SEM) of myelin signal density in the midline of the callosal body from treatment groups shown in **(A)**. ^*^p < 0.05, ^**^p < 0.01. One-way ANOVA with Tukey's multiple comparisons test and unpaired Student's *t*-test. One representative of 3 independent experiments is shown.

To assess the extent of myelination in *shiverer* mice with different treatments, myelin production was analyzed by MBP immunohistochemistry at day 90 postnatal (Figure 6A). In mice treated with PBS or FTY720, the absence of MBP leads to an almost complete lack of myelin stain in the brain (Figure 6A). In contrast, myelination of the brain was exhibited in the NSC or FTY720+NSC transplanted mice, which survived beyond 3–5 months. Remarkably, the degree of myelination, as well as the survival rate, was greater in FTY720-NSC co-treated mice than in mice only engrafted with NSCs (Figure 6). Together, these data indicate that engrafted NSCs can efficiently myelinate the hypomyelinated *shiverer* brain, and FTY720 significantly enhances this effect.

## Discussion

The appearance of myelin, and especially myelinogenesis postnatally, was a crucial step in vertebrate evolution and proper functioning of the mammalian CNS (Figlia et al., 2018). Impairment in myelin-producing cell (oligodendrocyte) development and subsequent myelination deficits appear as a common denominator of demyelinating diseases, ranging from pediatric leukodystrophies and cerebral palsy, to MS and white matter stroke (Goldman et al., 2012). Chronic demyelinating diseases also play a role in the pathogenesis of other neurological diseases, such as stroke, amyotrophic lateral sclerosis, and Alzheimer's disease (Höftberger and Lassmann, 2017). Accordingly, replacing lost oligodendrocytes by endogenous or exogenous progenitor cells, such as NSCs or OPCs, holds great promise as a therapeutic strategy (Goldman et al., 2012). However, stem cell therapy alone is limited for clinical and pathological improvement, especially in chronic demyelinating diseases, because of its relatively slow rate of oligodendrocyte differentiation (Franklin and Ffrench-Constant, 2008). It has been demonstrated that the primary cause of stem cell inefficiency is that the hostile, sustained inflammation stress and loss of trophic support weaken cell viability and differentiation capacity, and even cause intrinsic defects in transplanted NSCs or other multipotent stem cells in CNS lesions (Gao et al., 2014). Therefore, strategies targeted at suppressing CNS inflammation, converting a hostile environment for transplanted cells into a supportive one, and promoting OLG maturity, could be ideal for the treatment of chronic demyelinating diseases (Xiao et al., 2018).

In our previous study, we demonstrated an effective therapy combining FTY720 and transplanted NSCs that significantly alleviated the chronic stage of relapsing-remitting EAE (Zhang et al., 2017). The therapeutic effects correlated with not only the reduction of demyelination, promotion of remyelination of the EAE pathological process, inhibition of axonal degeneration and astrogliosis, but also with the efficient improvement of survival of transplanted NSCs and promotion of development of OPCs into OLGs *in vivo* (Zhang et al., 2017). Although these results are encouraging, there remained the question whether the neuroprotective effect with FTY720 and NSCs is a secondary consequence of the immunomodulatory activity in the inflamed CNS or could also be manifested under nonpathological conditions. Here we attempted to address this important question and investigate the direct effects of the combination therapy on myelinogenesis and oligodendrogenesis in the developing nervous system by using three myelination models without the involvement of immune responses.

Numerous studies *in vitro* had demonstrated that FTY720 induces OPC process extension and differentiation in every instance (Coelho et al., 2007; Jung et al., 2007; Miron et al., 2008a; Bieberich, 2011; Cui et al., 2014). Although cultures of oligodendrocyte lineage cells have been frequently used to study aspects of oligodendroglia cell biology, the obvious weakness of the *in vitro* cell culture system is that it does not accurately recapitulate the complex 3-D structure of tissue, and the cells behave “abnormally” in very contrived environments (Jarjour et al., 2012). An alternative is to use an *ex vivo* brain slice culture model, in which the myelination process happens within a multicellular environment that maintains physiological cell-cell interactions (Zhang et al., 2011). It can be used as an intermediate step between *in vitro* experiments and undertaking lengthy *in vivo* studies. In a previous study, Miron and colleagues (Miron et al., 2010) reported that FTY720 alone had no impact on myelination of brain slices under basal conditions. We also observed similar results in the single FTY720-treated group. However, when we treated brain slices from newborn mice with FTY720 plus NSCs, we found that FTY720 could drive NSCs to differentiate into oligodendrocytes and promote myelination significantly in an *ex vivo* brain slice culture model. In addition, NSCs may have trophic effects by releasing neurotrophic factors BDNF and GDNF, which alter the behavior of endogenous OPCs, promoting their maturation and myelination (De Feo et al., 2012). We speculated that these results were most likely due to S1P receptor expression or that the response of endogenous precursor cells (OPCs or NSCs) was modulated by the environment with inflammation stress, and the promising results of the combination therapy suggested that supplying FTY720 could induce a growth-promoting environment for endogenous OLG generation by transplanted NSCs, thus effectively promoting myelinogenesis.

We then further verified the potential of the combination therapy to promote postnatal myelinogenesis in healthy newborn mice as well as in *shiverer* mice with *MBP* gene deletion. The cumulative findings, obtained by both immunohistological and biochemical analyses, indicated that FTY720 treatment alone did not obviously affect myelination, and NSC treatment alone resulted in a slight, but significant, improvement. Notably, a remarkable increase in the number of myelin-encircled axons and CC1^+^ mature oligodendrocytes could be detected in the CNS of 14-day-old mice co-treated with FTY720 and NSCs, compared to the single- or PBS-treated littermates. The increased myelinogenesis that resulted from FTY720 and NSC co-treatment, obtained in this study in both healthy and genetically defective infant mice, is in accord with our previous findings, which indicated similar effects in mature EAE-induced mice (Zhang et al., 2017). Likewise, it was demonstrated that a novel mechanism of FTY720 action in NSC differentiation and remyelination. Taken together, our *in vivo* findings demonstrate that FTY720 and NSC co-treatment accelerated myelin development in the newborn mice at non-inflammatory conditions, thus confirming a direct, immunomodulation-independent effect for myelination.

The underlying mechanism by which FTY720 and NSCs affect myelinogenesis and oligodendrogenesis in the developing nervous system under nonpathological conditions was attributed to a neurotrophic effect, namely, a modulation of growth factor expression. In this study, an increase in the expression of BDNF, GDNF, T3, NT3, LIF, IGF1, IL6, SHH, NGF, and GDF11 was demonstrated following the co-treatment in the CNS of the infant mice, and among them the increase in BDNF and GDNF was more prominent than in the others. These neurotrophins had been shown to promote myelination and regeneration during development as well as under pathological conditions (Acosta et al., 2013), and the observed effects of FTY720 and NSCs on myelination during postnatal development may result in part from the increased expression of beneficial growth factors. Also, the improved sensorimotor function observed at day 21 postnatal, as manifested by the co-treatment group exhibiting a better performance in the rotating rod test than their PBS-injected littermates, could be attributed to the lasting benefit of these growth factors, which positively influence myelin development and behavioral manifestations in the developing CNS.

Although remyelination activities of FTY720 treatment in chemical-induced non-inflammatory and EAE-induced inflammatory demyelination models have been reported (Slowik et al., 2015; Yazdi et al., 2015; Zhang et al., 2017), two advantages of using healthy or *shiverer* infant mice over EAE and the cuprizone-induced demyelination model are (Windrem et al., 2008; Wang et al., 2013): (1) unlike EAE mice, these models have a minimal (or none at all) immunological component such as microglia activation in the CNS, with no involvement of infiltrating immune cells from the periphery; (2) unlike cuprizone-induced demyelination, the mouse models do not involve chemical toxicity to myelinating cells in the CNS. Taken together, the myelination models in this study provided evidence for the direct effect of FTY720 on NSC differentiation into oligodendrocytes, as well as that FTY720 promoted myelination by NSCs *in vivo*, in addition to its well-known immunomodulatory effects. The results also provide useful information on other neurological disorders with similar characteristics (minimal CNS inflammation and non-chemical-induced), such as neurodegenerative diseases.

## Author Contributions

YZ, XL, and G-XZ conceived and designed the experiments, analyzed data, and wrote the manuscript. YZ, X-YL, Z-QY, and XL carried out the experiments. C-GM and BC designed the experiments and interpreted the data. BC and AR co-supervised the study and wrote the paper. All authors read and approved the final manuscript.

### Conflict of Interest Statement

The authors declare that the research was conducted in the absence of any commercial or financial relationships that could be construed as a potential conflict of interest.

## Supplementary Material

Video 1 Seizure frequency of PBS-treated shiverer mice.

Video 2 Diminished seizure frequency and improved ambulation of shiverer mice treated with FTY720 in combination with transplanted NSCs.

## Abbreviations

BDNF: brain-derived neurotrophic factor
GDNF: glial cell line-derived neurotrophic factor
CNS: central nervous system
EAE: experimental autoimmune encephalomyelitis
FTY720: fingolimod
GFAP: glial fibrillary acid protein
MBP: myelin basic protein
MS: multiple sclerosis
NeuN: Neuronal Nuclei
NFH: neurofilament
NSCs: neural stem cells
OLGs: oligodendrocytes
OPCs: oligodendrocyte precursor cells
S1P: sphingosine 1-phosphate.
