# Corrigendum: Combination therapy with fingolimod and neural stem cells promotes functional myelination *in vivo* through a non-immunomodulatory mechanism

**DOI:** 10.3389/fncel.2022.1022297

**Published:** 2022-12-15

**Authors:** Yuan Zhang, Xin-Yu Lu, Ze-Qin Ye, Bogoljub Ciric, Cun-Gen Ma, Abdolmohamad Rostami, Xing Li, Guang-Xian Zhang

**Affiliations:** ^1^Department of Neurology, Thomas Jefferson University, Philadelphia, PA, United States; ^2^National Engineering Laboratory for Resource Development of Endangered Crude Drugs in Northwest China, The Key Laboratory of Medicinal Resources and Natural Pharmaceutical Chemistry, The Ministry of Education, Shaanxi Normal University, Xi'an, China; ^3^Department of Neurology, Institute of Brain Science, Shanxi Datong University Medical School, Datong, China

**Keywords:** fingolimod, neural stem cells, myelination, oligodendrocytes, combination therapy

In the published article, there was an error in Figure 2 as published. Due to a mistake made during figure preparation, there was an error in the CC1-GFP-stained image in the PBS and FTY720 group of Figure 2B. The corrected Figure 2 and its caption appears below.

**Figure 2:**
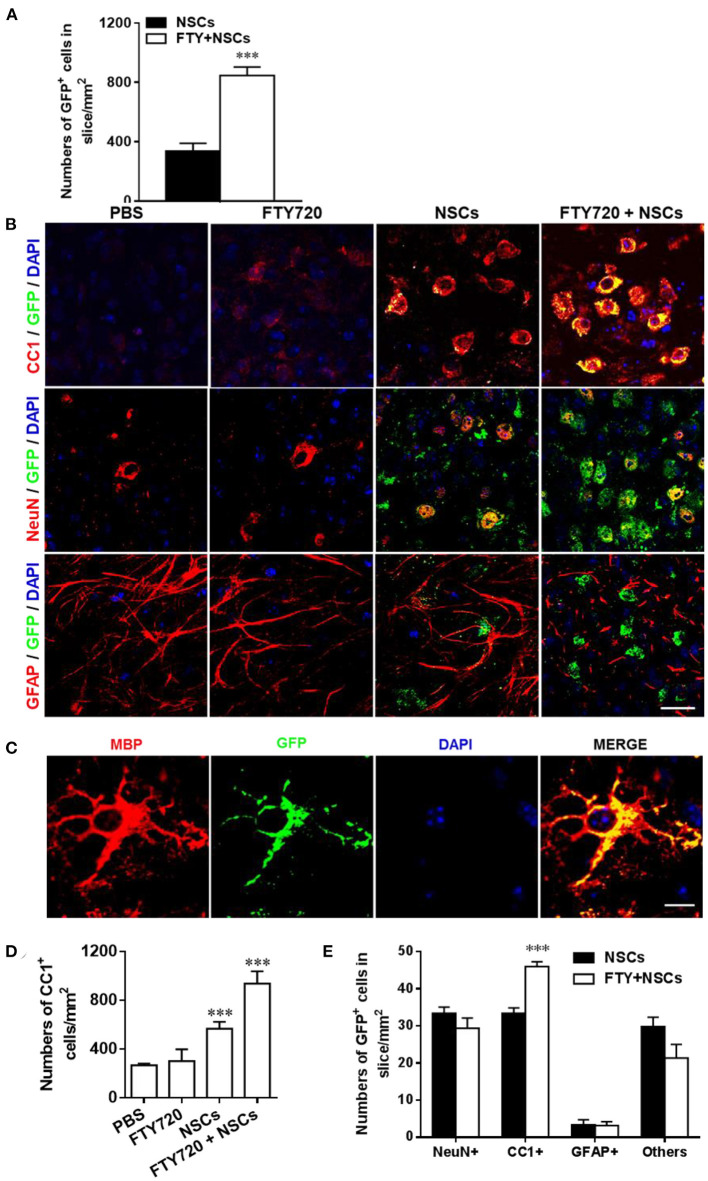
Impact of FTY720 plus NSC treatment on neural cell lineages in brain slice culture model. Brain slices obtained from newborn (P0-3) C57/Bl6 mouse pups were grown for 3 days in culture, and NSCs (2 μl, ~5 × 10^4^ cells /slice) and/or FTY720 (1 nM) were added for the subsequent 7 days; the slices were then fixed and immunostained. **(A)** Quantitative analysis of the number of transplanted NSCs (GFP+) in brain slices. **(B)** Immunofluorescence images of brain slices. Cells co-labeled with GFP and neural specific markers (red) were identified as differentiated cells derived from NSCs (yellow); cells positive only for neural-specific markers (red) were endogenous cells. CC1+: oligodendrocytes, NeuN+: neurons, GFAP+, astrocytes. Scale bar = 20 μm. **(C)** High-magnification confocal images show that GFP (green) was highly colocalized with the oligodendrocyte marker MBP (red). Scale bar = 10 μm. **(D)** Quantification of total CC1+ cell numbers. **(E)** Quantitative analysis of differentiation of transplanted NSCs in the CNS as shown in **(B)**. Symbols represent mean ± SD; *n* = 10 random areas per group. ^***^p < 0.001. One-way ANOVA with Tukey's multiple comparisons test and unpaired Student's *t*-test. One representative of 3 independent experiments is shown.

The authors apologize for this error and state that this does not change the scientific conclusions of the article in any way. The original article has been updated.

